# Editorial: Recent advancements in motor imagery

**DOI:** 10.3389/fnins.2023.1283413

**Published:** 2023-09-26

**Authors:** Kishor Lakshminarayanan, Deepa Madathil, Bhaskar Mohan Murari

**Affiliations:** ^1^School of Electronics Engineering, Vellore Institute of Technology, Vellore, India; ^2^Jindal Institute of Behavioural Sciences, O.P. Jindal Global University, Sonipat, Haryana, India

**Keywords:** electroencephalography, neurofeedback (NFB), tDCS, motor execution (ME), motor imagery

Motor imagery (MI) is a cognitive technique that has garnered increasing attention in recent years for its potential in enhancing various aspects of human performance, from motor learning and rehabilitation to sports training. MI involves mentally rehearsing physical actions without actual physical movement. Our topic on recent advancements in motor imagery has received a collection of articles that have explored innovative ways to optimize MI's effectiveness, and their findings are shedding new light on how this technique can be harnessed to unlock human potential.

Motor imagery has been shown to benefit largely when combined with other modalities of sensory-motor stimulation or feedback. Recent research has explored the effects of modalities such as transcranial direct current stimulation (tDCS) and neurofeedback (NFB) in terms of MI performance by the subjects as well as the cortical response. However, the combined effect of tDCS and NFB has been largely unexplored. A study by Sawai, Murata et al. investigated the impact of applying tDCS immediately prior to MI with NFB. Participants were divided into two groups: NFB and tDCS + NFB. Both groups completed 60 MI trials, with measurements of μ-event-related desynchronization (μ-ERD) and MI vividness taken before and after the training. The findings revealed that the tDCS + NFB group showed a significant increase in μ-ERD compared to the NFB-only group, indicating enhanced MI brain activity. Moreover, post-training, both groups exhibited increased MI vividness. This suggests that combining tDCS and NFB is more effective in improving MI abilities than using them separately.

Following their previous study, Sawai, Fujikawa et al. further examined the impact of NFB on combined MI and motor execution (ME) training. Sixteen participants were divided into two groups: MI-ME and MI-ME + NFB. Both groups performed 10 trials of a standing postural control task on an unstable board (ME) with nine MI trials in-between. Electroencephalogram (EEG) data were collected during the tasks. In the MI-ME + NFB group, participants received auditory feedback on their MI quality. Both groups showed reduced postural instability after the MI-ME training, but the MI-ME + NFB group had significantly less board sway, indicating enhanced learning when combining NFB with MI-ME.

Another form of stimulation similar to tDCS is functional electrical stimulation (FES), which has shown considerable promise in enhancing MI performance. With the suppression of the μ-rhythm in the EEG being a well-established indication of sensorimotor cortex activity during MI, a study by Yakovlev et al. investigated how FES affects the μ-rhythm suppression during MI. Thirteen healthy volunteers were recruited for the study. Three conditions were evaluated namely, MI, MI + FES, and FES. During the MI and MI + FES conditions, the participants were instructed to imagine right hand grasping. A 30-channel EEG was recorded during all three conditions. The results demonstrated that MI + FES resulted in a significantly larger μ-rhythm suppression compared to the other conditions. The results imply that peripheral electrical stimulation has a direct influence on brain activity, particularly when paired with MI. The study also demonstrated the potential advantages of using FES in MI-based interventions to improve cortical activation.

Advancing the research on other forms of stimulation similar to tDCS and FES to enhance MI, a study conducted by Ramu and Lakshminarayanan evaluated how vibrotactile stimulation affects the performance of MI in healthy adults. Ten right-handed participants engaged in MI tasks involving finger movements while receiving brief vibratory stimulation to their right-hand finger-pads right before each MI trial. Results showed significantly increased event-related desynchronization (ERD) in the sensorimotor cortex during MI when participants received vibration compared to when they didn't. Digit classification accuracy, assessed via artificial neural networks, was notably higher with vibration. This study suggests that short vibrotactile stimuli effectively enhance MI-related brain activity and improve digit classification within a brain-computer interface (BCI)-based MI system.

Delving into the other realms of MI, a study by Gäumann et al. aimed at assessing MI engagement in stroke patients by analyzing eye movement recordings. Twenty-one stroke patients participated in the study, undergoing assessments including the Kinaesthetic and Visual Imagery Questionnaire (KVIQ-10), body rotation, and mental chronometry tasks. Smart eyeglasses equipped with electrooculography (EOG) electrodes recorded eye movements, alongside heart rate (HR), oxygen saturation (SpO2), and EEG data. Two sessions compared self-explanation (SE) performance during MI, physical exercise (PE), and rest to assess SE's psychometric properties. Quantifying MI participation in stroke patients is challenging, but HR proved reliable. Interestingly, eye movements during SE in MI didn't resemble those during PE, likely due to task complexity.

Although MI performance and even engagement have been largely evaluated from cortical activity, the effect of MI on actual limb physiology has been largely unexplored. In a study conducted by Piveteau et al., the researchers aimed to explore the impact of MI on enhancing muscle strength, particularly in large limb muscles. They recruited 75 individuals and divided them into three groups, all of which engaged in a back squat workout. The first group practiced MI specifically related to the back squat, while the second group practiced MI of a different lower limb exercise (the deadlift) during rest intervals between trials. The control group, on the other hand, spent the same amount of time engaged in a neutral cognitive activity. The results revealed that the two groups that practiced MI, especially focusing on the back squat, demonstrated greater improvements in their back squat performance compared to the control group. The results suggests that MI can indeed have a positive impact on enhancing strength training, particularly in multi-joint dynamic exercises involving the lower limbs.

Recent advancements in motor imagery research, utilizing technologies like tDCS, NFB, vibrotactile stimulation, and motor execution, are revolutionizing our understanding of mental influence on physical performance. These advancements hold promise for motor learning, rehab, and sports training. Combining functional electrical stimulation and eye movement tracking and HR with MI offers new paths for neurorehabilitation and stroke recovery. As these research frontiers continue to expand, it is imperative for practitioners, clinicians, and educators to remain open to the evolving possibilities that motor imagery offers. These recent advancements herald a future where the mind's influence on the body is more effectively harnessed, ultimately enhancing the quality of life for countless individuals.

## Author contributions

KL: Writing—original draft. DM: Writing— review and editing. BM: Writing—review and editing.

